# Efbemalenograstim alfa not inferior to pegfilgrastim in providing neutrophil support in women with breast cancer undergoing myelotoxic chemotherapy: results of a phase 2 randomized, multicenter, open-label trial

**DOI:** 10.1007/s00520-023-08260-x

**Published:** 2024-01-09

**Authors:** John Glaspy, Igor Bondarenko, Dmitrii Krasnozhon, Dean Rutty, Jianmin Chen, Yanyan Fu, Shufang Wang, Qingsong Hou, Simon Li

**Affiliations:** 1 UCLA School of Medicine, 100 UCLA Medical Plaza, Suite 550, Los Angeles, CA 90095-6956 USA; 2 Dnepropetrovsk Medical Academy, Dnepropetrovsk, Ukraine; 3 GBUZ LOOD Surgery Department, Leningrad Regional Oncology Center, Saint Petersburg, Russia; 4 Everest Clinical Research, Markham, Ontario Canada; 5 Evive Biotechnology (Shanghai) Ltd, Shanghai, China

**Keywords:** Efbenmalenograstim alfa, Neutropenia, G-CSF, Breast cancer

## Abstract

**Purpose:**

Evaluate the safety and efficacy of efbemalenograstim alfa for neutrophil support in breast cancer patients undergoing myelosuppressive chemotherapy in a phase 2, dose-finding, open-label study (NCT01648322, ClinicalTrials.gov, 2012–07-19).

**Methods:**

232 patients received up to 4 cycles of chemotherapy, 141 patients with docetaxel + cyclophosphamide (TC) and 91 patients with docetaxel + doxorubicin + cyclophosphamide (TAC). Patients were randomized to efbemalenograstim alfa (80, 240, or 320 µg/kg [TC]; 240 or 320 µg/kg [TAC]) or pegfilgrastim (6 mg) on Day 2 of each cycle.

**Results:**

Efbemalenograstim alfa was non-inferior to pegfilgrastim in duration of moderate and severe neutropenia (absolute neutrophil count [ANC] < 1.0 × 10^9^/L) in TAC Cycle 1 (mean [SD] of 2.1 [1.58] and 2.1 [1.46] days for 240 µg/kg and 320 µg/kg efbemalenograstim alfa, respectively, and 1.8 [1.28] days for pegfilgrastim), with a difference (95% CI) of 0.3 (-0.4, 1.1) days. ANC nadir occurred between Days 7–8 of TAC Cycle 1, with mean [SD] of 0.68 [1.064], 0.86 [1.407] and 0.78[1.283] × 10^9^/L for 240 µg/kg, 320 µg/kg efbemalenograstim alfa and pegfilgrastim, respectively. Time to ANC recovery post nadir (defined as an ANC > 2.0 × 10^9^/L after the expected ANC nadir) was 2.0–2.4 and 1.9 days for TAC patients treated with efbemalenograstim alfa and pegfilgrastim, respectively. No significant difference was found between any dose of efbemalenograstim alfa and pegfilgrastim in TAC Cycle 1 for incidence of moderate to severe neutropenia (76%-77% of patients) or incidence of severe neutropenia (ANC < 0.5 × 10^9^/L; 63%-72%). Efbemalenograstim alfa exhibited similar safety profile to pegfilgrastim. Febrile neutropenia occurred in 4 (1.8%) patients, 2 patients each for 320 µg/kg efbemalenograstim alfa and pegfilgrastim, with no event considered related to study drug.

**Conclusion:**

Efbemalenograstim alfa was comparable to pegfilgrastim in efficacy and safety.

**ClinicalTrials.gov identifier:**

NCT01648322.

## Introduction

Neutropenia is a common side effect associated with myelosuppressive chemotherapy that negatively impacts patient safety due to an increased risk of infection, and delays and/or reduction of dose in chemotherapy treatment. Adjunct to the safety concerns are a rise in health care expenses associated with treating these complications, such as more frequent and/or longer hospital stays and increased drug costs [1, 2]. Accordingly, the current standard clinical practice often employs the use of recombinant granulocyte colony stimulating factors (G-CSFs) in conjunction with myelosuppressive chemotherapy with significant infection risks, to help reducing the duration of neutropenia and the risk of infection.

Human G-CSF is a hematopoietic growth factor that is present in low to undetectable levels in blood plasma [3]. Binding of G-CSF to its cell surface receptor (G-CSFR) causes the receptor to homodimerize, resulting in the activation of the Janus kinase-signal transducer and activator of transcription pathway that ultimately leads to proliferation and differentiation of neutrophil precursors and activation of mature neutrophils. Plasma concentrations of G-CSF increase in response to neutropenia or bacterial infection, stimulating the activation and production of neutrophils, and then decline with recovery of absolute neutrophil counts (ANCs) to normal levels [3–5].

Recombinant G-CSFs currently approved for use in the management of chemotherapy-induced neutropenia include filgrastim (Neupogen®), pegfilgrastim (Neulasta®, Neulastim®), lenograstim (Granocyte™), and eflapegrastim (Rolvedon™) recently approved by FDA. Filgrastim and lenograstim both necessitate daily injections to impact neutropenia, while pegfilgrastim is a pegylated G-CSF and eflapegrastim is a pegylated IgG_4_ F_C_ fusion protein, both administered once-per chemotherapy cycle, typically 24 h after chemotherapy treatment. Although PEGylation (PEG) of filgrastim is successful in decreasing renal clearance and prolonging its half-life, many patients have anti-PEG antibodies due to exposure to PEG from various products (laxatives, cosmetics) [6]. The presence of anti-PEG antibodies in patients has been associated with a loss of therapeutic efficacy and an increase in adverse reactions [7].

Efbemalenograstim alfa is a novel recombinant G-CSF that fuses human G-CSF with human IgG_2_-F_c_ which is dimeric in form. It is administered once-per chemotherapy cycle, making it a potential alternative to pegfilgrastim that may also be a more potent activator of G-CSFR. In vivo studies have suggested that efbemalenograstim alfa may generate faster neutrophil recovery and reduce the severity of cyclophosphamide-induced neutropenia in monkeys when compared to filgrastim or pegfilgrastim [8]. These data support the conclusion that efbemalenograstim alfa may be non-inferior or superior to pegfilgrastim as once per cycle myeloid growth factor support for myelosuppressive chemotherapy.

The purpose of this clinical trial is to evaluate the efficacy and safety of three doses of efbemalenograstim alfa compared to pegfilgrastim in aiding ANC recovery post-treatment during a myelotoxic chemotherapy.

## Methods

### Study patients

Eligible patients were females 18–75 years of age, diagnosed with Stage I-IV invasive breast cancer with an Eastern Cooperative Oncology Group performance status ≤ 2 and scheduled for TC (75 mg/m^2^ Taxotere® [docetaxel] + 600 mg/m^2^ cyclophosphamide) or TAC (75 mg/m^2^ Taxotere® [docetaxel] + 50 mg/m^2^ doxorubicin + 600 mg/m^2^ cyclophosphamide) chemotherapy. Patients must have had white blood cell (WBC) count ≥ 4.0 × 10^9^/L, hemoglobin ≥ 11.5 g/dL, platelet count ≥ 150 × 10^9^/L, and adequate renal, hepatic, and cardiac function. Key exclusion criteria included: prior treatment (within 6 weeks) with a G-CSF or a drug that may potentiate release of neutrophils, recent radiation therapy (within 4 weeks), prior chemotherapy (within 1 year), and prior bone marrow or stem-cell transplantation. Patients with a history of prior malignancy other than breast cancer may have entered the study if the malignancy was in remission.

### Study design

This was a phase 2, open-label, active controlled, dose-finding clinical study that occurred at 22 sites in the United States, Russia, and Ukraine (ClinicalTrials.gov identifier: NCT01648322). Initial patients enrolled in this study received TC chemotherapy. Following an interim analysis, the incidence of severe neutropenia with TC chemotherapy was too low to adequately explore the study objectives and the protocol was amended to limit chemotherapy to the TAC regimen. TC and TAC chemotherapy were administered by intravenous injection on the first day of each 21-day chemotherapy cycle for up to 4 cycles. Approximately 24 h after completion of chemotherapy, patients received a subcutaneous injection of their randomized study treatment. Patients receiving TC chemotherapy were randomized 1:1:1:1 to 80, 240, or 320 µg/kg efbemalenograstim alfa or 6 mg pegfilgrastim. Patients receiving TAC chemotherapy were randomized 1:1:1 to 240 or 320 µg/kg efbemalenograstim alfa or 6 mg pegfilgrastim. Randomization was performed by an Interactive Web-based Response System and was stratified by country/region to reduce regional bias. Patients remained on their randomized study drug dose and chemotherapy regimen for each chemotherapy cycle. To continue to the next chemotherapy cycle, patients were required to have a hemoglobin level ≥ 11.5 g/dL, WBC > 4.0 × 10^9^/L, and platelet count > 100 × 10^9^/L.

ANC profile post-chemotherapy was tracked with daily blood draws in Cycle 1 and blood draws every other day in Cycles 2–4 until ANCs reached ≥ 2.0 × 10^9^/L, post nadir, and then every 3 days until the next chemotherapy cycle. If patients had an ANC < 0.5 × 10^9^/L for 2 consecutive visits, daily blood draws occurred until the level returned to > 0.5 × 10^9^/L. Patients with an ANC < 0.5 × 10^9^/L for 5 consecutive days were withdrawn from the study.

### Efficacy and safety assessments

The primary efficacy endpoint was the duration of moderate and severe neutropenia during Cycle 1, defined as the number of days in which the patient had an ANC < 1.0 × 10^9^/L during Cycle 1.

Secondary efficacy endpoints included the duration of moderate and severe neutropenia (ANC < 1.0 × 10^9^/L) in Cycles 2–4, the duration of severe neutropenia (ANC < 0.5 × 10^9^/L) in each cycle, the incidence rates of febrile neutropenia in each cycle, the time in days to ANC recovery post nadir (recovery defined as an ANC ≥ 2.0 × 10^9^/L after the expected ANC nadir) in each cycle, and the depth of the ANC nadir in each cycle.

Safety assessments included adverse events (AEs), serious adverse events (SAEs), clinical laboratory parameters (hematology, blood chemistry, and urinalysis), vital signs, electrocardiograms (ECGs), and physical examinations. AEs of special interest included febrile neutropenia, injection site reactions, and infections.

AEs and SAEs were collected from the date of informed consent until 30 days after the completion of the study. AEs were classified by system organ class and preferred term according to the Medical Dictionary for Regulatory Activities Version 17.1. The severity of AEs was graded based on the National Cancer Institute Common Terminology Criteria for Adverse Events v4.0. Relationships between AEs and the study treatments were determined by the site Investigators.

All laboratory tests used for statistical analyses were performed by a designated central laboratory to ensure consistent measurements throughout the study duration.

### Statistical methods

For the primary efficacy analysis, non-inferiority was determined by comparing the duration of moderate and severe neutropenia between each dose of efbemalenograstim alfa and pegfilgrastim in Cycle 1 within 12 days of chemotherapy treatment. Non-inferiority to pegfilgrastim was declared if the upper limit of the 2-sided Wald confidence interval for the difference in mean duration was ≤ 2 days. If efbemalenograstim alfa was found to be non-inferior to pegfilgrastim, superiority testing was to be performed with superiority claimed if the upper limit of the 2-sided Wald CI was < 0 days.

Based on the non-inferiority margin of 2 days and a common standard deviation of 1.5 days with 90% power, the study sample size was initially calculated as 50 patients per group (200 patients total) under the TC chemotherapy regimen. Following the change in chemotherapy regimen from TC to TAC and the exclusion of the lowest F-627 dose (after 141 patients were randomized), an additional 32 patients were added to the overall sample size (232 patients total) allowing 91 patients to be randomized to the remaining 3 treatment arms under the TAC chemotherapy regimen.

The primary analysis population for all efficacy analyses was the Per Protocol (PP) analysis set, which included all patients in the Intent-to-Treat (ITT) analysis set who received study treatment, were eligible and compliant, and were without major protocol deviations during the first cycle of treatment. Safety analyses were performed on all enrolled patients receiving any study treatment.

## Results

### Study patients

**Fig. 1:**
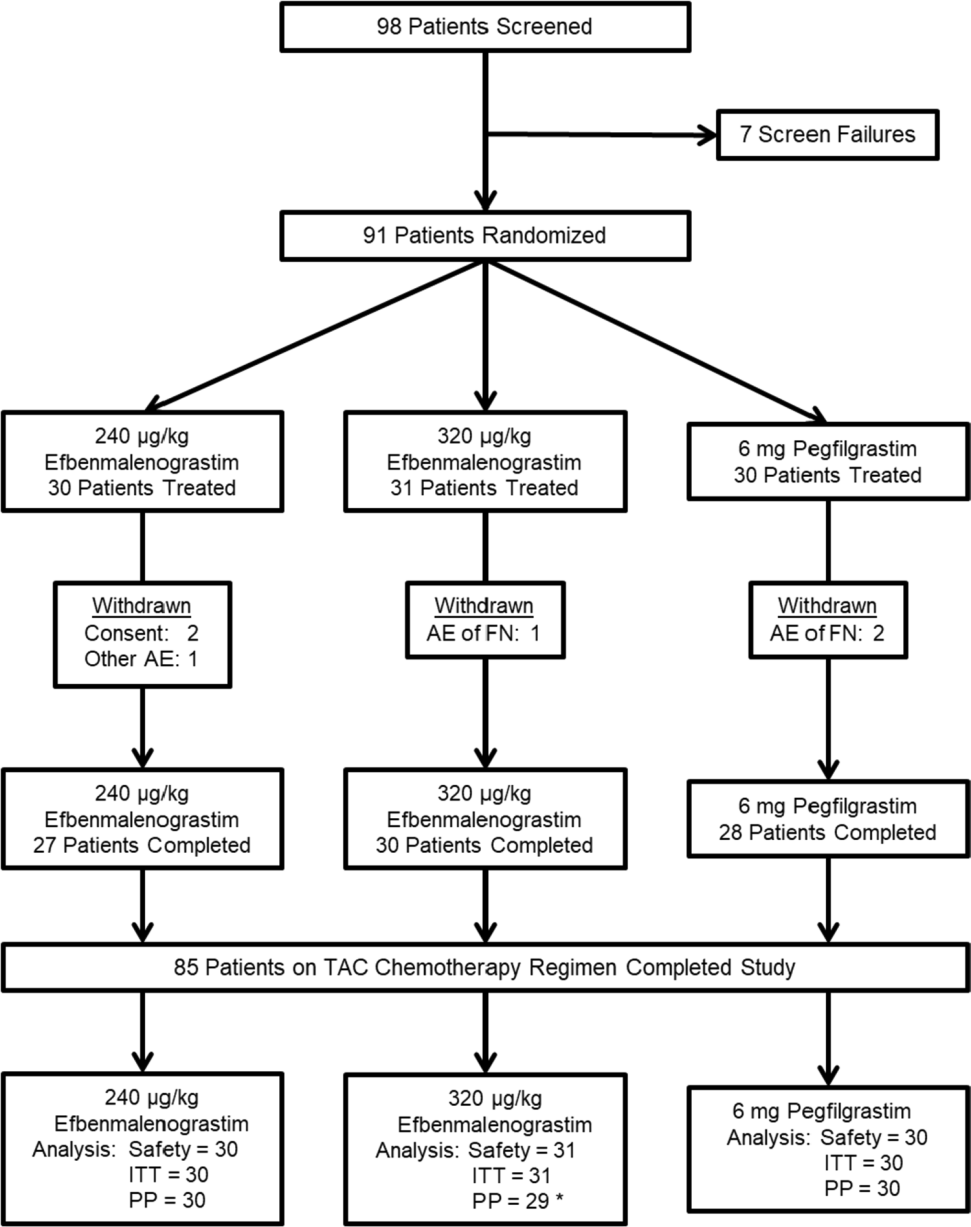
Patient flow for TAC chemotherapy population. AE = adverse event; FN = febrile neutropenia; ITT = Intent-to-Treat; PP = Per Protocol; TAC = Taxotere.® [docetaxel] + doxorubicin + cyclophosphamide. * 2 patients were excluded from the PP population due to lack of absolute neutrophil count data in chemotherapy cycle 1

There were 249 patients screened in this study, with 232 patients randomized and treated (91 for TAC chemotherapy and 141 for TC chemotherapy) at 22 sites in the United States, Russia, and Ukraine. A total of 216 patients (85 for TAC chemotherapy, 131 for TC chemotherapy) completed the study (Fig. 1 [TAC chemotherapy], Online Resource 1 [TC chemotherapy]). Patients withdrawn due to febrile neutropenia events included 3 treated with TAC chemotherapy and 1 treated with TC chemotherapy.

Randomized female patients were predominantly Caucasian ranging in age between 18 and 74 years, with the majority having Stage II or III breast cancer (approximately 80%). Other baseline and disease status characteristics are shown in Table 1 (TAC chemotherapy) and Online Resource 2 (TC chemotherapy). Efbemalenograstim alfa and pegfilgrastim treatment arms had similar baseline and disease characteristics in each of the chemotherapy populations.

**Table 1:** Baseline demographic and disease status for patients in TAC chemotherapy population

	Efbemalenograstim alfa		Pegfilgrastim 6 mg *N* = 30
	240 μg/kg *N* = 30	320 μg/kg *N* = 31	
Age, mean (range), years	48.2 (28, 72)	46.3 (26, 66)	48.2 (26, 65)
Reproductive status			
Childbearing potential	20 (66.7)	16 (51.6)	16 (53.3)
Non-childbearing potential	10 (33.3)	15 (48.4)	14 (46.7)
Race			
Caucasian	30 (100.0)	31 (100.0)	30 (100.0)
Country			
Ukraine	14 (46.7)	15 (48.4)	14 (46.7)
Russia	16 (53.3)	16 (51.6)	16 (53.3)
USA	0	0	0
ECOG performance status			
0	20 (66.7)	22 (71.0)	17 (56.7)
1	10 (33.3)	9 (29.0)	13 (43.3)
Cancer stage at screening			
I	4 (13.3)	5 (16.1)	5 (16.7)
II	13 (43.3)	17 (54.8)	10 (33.3)
III	9 (30.0)	6 (19.4)	13 (43.3)
IV	4 (13.3)	3 (9.7)	2 (6.7)
Prior surgery for breast cancer	26 (86.7)	26 (83.9)	26 (86.7)
Prior systemic therapy	2 (6.7)	3 (9.7)	3 (10.0)
Prior radiation therapy	4 (13.3)	3 (9.7)	7 (23.3)

Data presented as *n* (%) unless otherwise indicated

*ECOG* Eastern cooperative oncology group; *TAC* Taxotere® [docetaxel] + doxorubicin + cyclophosphamide

### Efficacy

#### Duration of neutropenia in chemotherapy cycle 1

For the TAC chemotherapy population, the mean (SD) duration of moderate and severe neutropenia in Cycle 1 was 2.1 (1.58) and 2.1 (1.46) days for 240 µg/kg and 320 µg/kg efbemalenograstim alfa, respectively, and 1.8 (1.28) days for pegfilgrastim (Table 2). Both doses of efbemalenograstim alfa were non-inferior to pegfilgrastim with a difference (95% CI) of 0.3 (-0.4, 1.1) days for each dose of efbemalenograstim alfa compared to pegfilgrastim. Neither dose of efbemalenograstim alfa was superior to pegfilgrastim. The duration of severe neutropenia (ANC < 0.5 × 10^9^/L) with efbemalenograstim alfa and pegfilgrastim treatment following TAC chemotherapy was comparable (1.4–1.5 days and 1.1 days, respectively). For the TC chemotherapy population, similar results supporting non-inferiority of efbemalenograstim alfa to pegfilgrastim were observed (Online Resource 3).

**Table 2:** Duration of neutropenia in TAC chemotherapy cycle 1

	Efbemalenograstim alfa		Pegfilgrastim 6 mg *N* = 30
	240 µg/kg *N* = 30	320 µg/kg *N* = 29	
Moderate and severe neutropenia (ANC < 1.0 × 10^9^/L)			
Duration, days			
Mean (SD)	2.1 (1.58)	2.1 (1.46)	1.8 (1.28)
Median (range)	2.0 (0, 6)	2.0 (0, 4)	2.0 (0, 4)
Difference vs. pegfilgrastim (95% CI)	0.3 (−0.4, 1.1)	0.3 (−0.4, 1.1)	
Non-inferior to pegfilgrastim?	Yes	Yes	
Superior to pegfilgrastim?	No	No	
Severe neutropenia (ANC < 0.5 × 10^9^/L)			
Duration, days			
Mean (SD)	1.5 (1.55)	1.4 (1.15)	1.1 (1.01)
Median (range)	1.0 (0, 6)	2.0 (0, 4)	1.0 (0, 3)
Difference vs. pegfilgrastim (95% CI)	0.4 (−0.2, 1.0)	0.4 (−0.3, 1.0)	

*ANC* Absolute neutrophil count; *CI* Confidence interval; *SD* Standard deviation; *TAC* Taxotere® [docetaxel] + doxorubicin + cyclophosphamide

#### Depth of nadir and time to recovery in chemotherapy cycle 1

**Fig. 2:**
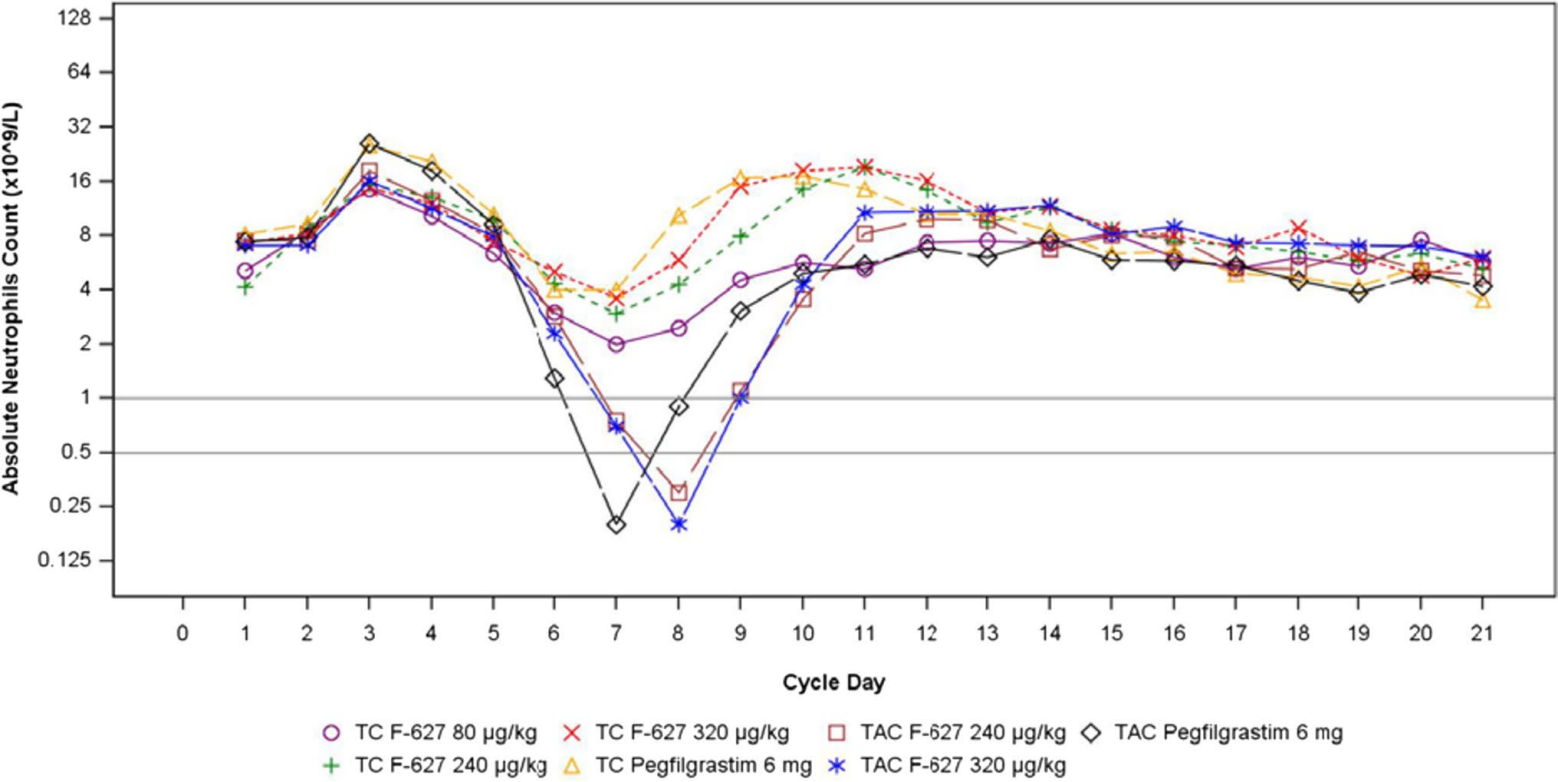
Median absolute neutrophil count during chemotherapy cycle 1. F-627 = Efbenmalenograstim alfa; TAC = Taxotere® [docetaxel] + doxorubicin + cyclophosphamide; TC = Taxotere® [docetaxel] + cyclophosphamide

During chemotherapy Cycle 1, ANC peaked at Day 3 and reached nadir between Days 7 and 8 before beginning to recover (Fig. 2). Depth of ANC nadir was lower in patients receiving TAC chemotherapy (0.68–0.86 × 10^9^/L) compared to TC chemotherapy (2.09–3.05 × 10^9^/L) patients in Cycle 1 (Table 3). Time to recovery post nadir (ANC > 2.0 × 10^9^/L) was 2.0–2.4 days and 1.9 days for patients treated with efbemalenograstim alfa and pegfilgrastim, respectively, in the TAC chemotherapy population. For patients receiving TC chemotherapy, time to recovery post nadir was 1.1 days for 80 µg/kg efbemalenograstim alfa and 0.4–0.6 days for the higher doses of efbemalenograstim alfa and pegfilgrastim (Table 3). ANC returned to baseline levels on approximately Day 10 with both agents.

**Table 3:** Incidence of neutropenia in chemotherapy cycle 1 and time to recovery post nadir

	Efbemalenograstim alfa			Pegfilgrastim 6 mg
	80 µg/kg	240 µg/kg	320 µg/kg	
TAC chemotherapy population		*N* = 30	*N* = 29	*N* = 30
ANC < 1.0 × 10^9^/L, n/N (%)		23/30 (76.7)	22/29 (75.9)	23/30 (76.7)
Difference vs. pegfilgrastim (*P*)^a^		0.0 (1.0000)	−0.8 (1.0000)	
ANC < 0.5 × 10^9^/L, n/N (%)		20/30 (66.7)	21/29 (72.4)	19/30 (63.3)
Difference vs. pegfilgrastim (*P*)^a^		3.3 (1.0000)	9.1 (0.5796)	
ANC depth of nadir, mean (SD), × 10^9^/L		0.68 (1.064)	0.86 (1.407)	0.78 (1.283)
Days to recovery post nadir, mean (SD)^b^		2.4 (1.56)	2.0 (1.27)	1.9 (1.20)
Difference vs. pegfilgrastim (95% CI)		0.4 (−0.3, 1.1)	0.0 (−0.7, 0.7)	
TC chemotherapy population	*N* = 35	*N* = 37	*N* = 31	*N* = 35
ANC < 1.0 × 10^9^/L, n/N (%)	10/35 (28.6)	10/37 (27.0)	6/31 (19.4)	7/35 (20.0)
Difference vs. pegfilgrastim (*P*)^a^	8.6 (0.5781)	7.0 (0.5830)	−0.6 (1.000)	
ANC < 0.5 × 10^9^/L, n/N (%)	4/35 (11.4)	7/37 (18.9)	5/31 (16.1)	3/35 (8.6)
Difference vs. pegfilgrastim (*P*)^a^	2.9 (1.0000)	10.3 (0.3092)	7.6 (0.4591)	
ANC depth of nadir, mean (SD), × 10^9^/L	2.09 (1.525)	2.41 (1.856)	3.00 (2.608)	3.05 (2.309)
Days to recovery post nadir, mean (SD)^b^	1.1 (1.28)	0.6 (0.95)	0.5 (0.81)	0.4 (0.60)
Difference vs. pegfilgrastim (95% CI)	0.7 (0.2, 1.1)	0.3 (−0.2, 0.7)	0.2 (−0.3, 0.6)	

*ANC* Absolute neutrophil count; *CI* Confidence interval; *SD* Standard deviation; *TAC* Taxotere® [docetaxel] + doxorubicin + cyclophosphamide; *TC* Taxotere® [docetaxel] + cyclophosphamide

^a^ Fisher’s Exact test

^b^ Days to recovery post nadir was a return of ANC to > 2.0 × 10^9^/L

#### Incidence of neutropenia in chemotherapy cycle 1

The incidence of moderate to severe neutropenia in Cycle 1 was 76%-77% of patients receiving TAC chemotherapy across all treatment arms and ranged between 19%-29% of patients receiving TC chemotherapy (Table 3). No significant difference in the incidence of moderate to severe neutropenia or severe neutropenia was observed between any dose of efbemalenograstim alfa and pegfilgrastim in Cycle 1 for both chemotherapy populations. Due to the low incidence of neutropenia and depth of ANC nadir observed with TC chemotherapy during study conduct, particularly moderate to severe neutropenia, the chemotherapy regimen was changed to TAC and the enrolment was extended to better observe differences between the treatment arms. Efficacy results obtained for both patient populations were similar.

#### Duration and incidence of neutropenia in chemotherapy cycles 2–4

Subsequent chemotherapy cycles exhibited a similar pattern of change for ANCs (Online Resource 4). Duration of moderate and severe neutropenia in each of Cycles 2–4 was shorter compared to Cycle 1, ranging between 0.6–1.6 days and 0.0–0.4 days for the TAC and TC chemotherapy populations, respectively, with no significant differences between efbemalenograstim alfa and pegfilgrastim (Online Resource 5). In the TAC chemotherapy population, duration of moderate or severe neutropenia in Cycles 3 and 4 was approximately half as long in patients treated with pegfilgrastim as compared to those treated with efbemalenograstim alfa; however, this difference was not statistically significant. Fewer patients experienced moderate or severe neutropenia in Cycles 2–4 compared to Cycle 1, ranging between 22–56% of patients in the TAC chemotherapy population (Online Resource 6) and 0–11.8% of patients in the TC chemotherapy population (data not shown). For the TAC chemotherapy population in Cycles 2 and 3, numerically higher incidence rates of moderate neutropenia were observed in patients treated with efbemalenograstim alfa as compared to pegfilgrastim; however, the differences were not statistically significant. Notably, a statistically higher incidence rate was observed in Cycle 4 for both 240 and 320 µg/kg efbemalenograstim alfa (56% [*p* = 0.0217] and 54% [*p* = 0.0261], respectively), compared to pegfilgrastim (22%; Online Resource 6).

In the TAC chemotherapy population, the incidence rates of severe neutropenia in Cycles 2–4 were comparable between the efbemalenograstim alfa and pegfilgrastim treatment arms, with no statistical differences in any cycle (Online Resource 6). The mean duration of severe neutropenia for the TAC chemotherapy population ranged between 0.2–0.8 days with efbemalenograstim alfa and 0.3–0.6 days with pegfilgrastim (data not shown).

### Safety and tolerability

The majority (91%-94%) of patients, including both TAC and TC chemotherapy patients, experienced a treatment-emergent AE, with comparable rates observed across treatment arms (Table 4). Treatment-related AEs were experienced by 29%-37% of efbemalenograstim alfa patients and 37% of pegfilgrastim patients. Few patients experienced an event leading to discontinuation from the study. The most common AEs across treatment groups included alopecia (68%-80%), nausea (33%-49%), and asthenia (19%-32%). The incidence of neutropenia was 17%-37% for efbemalenograstim alfa and 26% for pegfilgrastim. Febrile neutropenia occurred in 4 (1.8%) patients overall, 2 patients each for 320 µg/kg efbemalenograstim alfa and pegfilgrastim, with no event considered related to study treatment.

**Table 4:** Frequency of treatment emergent adverse events

Patients with treatment emergent adverse event, n (%)	Efbemalenograstim alfa			Pegfilgrastim 6 mg *N* = 65
	80 μg/kg *N* = 35	240 μg/kg *N* = 67	320 μg/kg *N* = 65	
Any TEAE	32 (91.4)	63 (94.0)	61 (93.8)	59 (90.8)
Treatment-related TEAE	10 (28.6)	21 (31.3)	24 (36.9)	24 (36.9)
AE leading to discontinuation	1 (2.9)	1 (1.5)	2 (3.1)	3 (4.6)
Any SAE	0	1 (1.5)	1 (1.5)	4 (6.2)
Febrile neutropenia	0	0	2 (3.1)	2 (3.1)
TEAEs > 10% in any treatment group:				
Alopecia	27 (77.1)	49 (73.1)	44 (67.7)	52 (80.0)
Nausea	17 (48.6)	22 (32.8)	23 (35.4)	28 (43.1)
Asthenia	11 (31.4)	13 (19.4)	21 (32.3)	20 (30.8)
Neutropenia	6 (17.1)	25 (37.3)	21 (32.3)	17 (26.2)
Bone pain	7 (20.0)	11 (16.4)	11 (16.9)	12 (18.5)
Fatigue	5 (14.3)	3 (19.4)	12 (18.5)	10 (15.4)
Leukopenia	3 (8.6)	12 (17.9)	8 (12.3)	7 (10.8)
Diarrhea	3 (8.6)	4 (6.0)	4 (6.2)	7 (10.8)
Headache	5 (14.3)	5 (7.5)	7 (10.8)	4 (6.2)

*SAE* Serious adverse event; *TEAE* Treatment-emergent adverse event

No deaths were reported and the incidence of SAEs was low with 6 (2.6%) patients reporting 10 events. The overall rate of SAEs was lower in patients treated with 240 or 320 µg/kg efbemalenograstim alfa (1.5%; 1 and 2 events in 1 patient each, respectively) compared to pegfilgrastim (6.2%; 7 events in 4 patients). The most common SAEs were febrile neutropenia (3 patients; 2 treated with pegfilgrastim and 1 with 320 µg/kg efbemalenograstim alfa) and toxic hepatitis (2 patients; 1 patient each treated with pegfilgrastim and 320 µg/kg efbemalenograstim alfa). The lengths of hospital stay for the 3 febrile neutropenia patients were 6 days (320 µg/kg efbemalenograstim alfa group), 7 days (Pegfilgrastim group), and 6 days (Pegfilgrastim group), respectively. Other SAEs, reported in 1 patient each included acute pancreatitis (pegfilgrastim group), gastroenteritis (pegfilgrastim group), acute cholecystitis (pegfilgrastim group), pneumonia (240 µg/kg efbemalenograstim alfa group), and hypersensitivity vasculitis (pegfilgrastim group).

Along with febrile neutropenia, injection site reactions and infections were events of special interest. Injection site reactions occurred in 3 (1.3%) patients receiving TC chemotherapy. Infections occurred in 14 (6.0%) patients overall, 3 (8.6%), 3 (4.5%), and 2 (3.1%) patients treated with 80, 240, and 320 µg/kg efbemalenograstim alfa, and 6 (9.2%) patients treated with pegfilgrastim. The most common infections, occurring in 2 patients each, were pneumonia, rhinitis, viral respiratory tract infection, and viral infection.

Laboratory values out of the normal range were observed for hematology parameters (neutrophils and leukocytes) and blood chemistry parameters (aspartate aminotransferase, alanine aminotransferase, gamma glutamyl transferase, lactate dehydrogenase); there were few differences in incidences of clinically significant abnormalities noted between treatment arms and no cycle- or dose-dependent trends were observed. There were no patients with clinically significant ECG findings and no AEs for abnormal ECGs were documented.

The overall safety profile of efbemalenograstim alfa observed in this study was comparable to pegfilgrastim with no patterns or trends of concern noted.

## Discussion

The risk of chemotherapy-induced neutropenia is influenced by patient-specific traits, such as age, co-morbidities and disease status; however, the level of myelotoxicity associated with a specific chemotherapy regimen is also important. A recently published prospective real-world study demonstrated that the incidence of febrile neutropenia was low, with a high incidence in cycle 1 and a decrease in the subsequent cycles, in patients receiving chemotherapy with intermediate risk of febrile neutropenia, but not G-CSF as primary prophylaxis [9]. Most of the current cancer regimens are associated with a 10–20% risk of neutropenia and breast cancer patients treated with a dose-dense anthracycline/taxane-based regimen, such as TAC, have > 20% risk of developing febrile neutropenia [10]. To help mitigate this safety risk, prophylactic use of G-CSF is recommended when the risk of febrile neutropenia, determined by considering patient factors and the chemotherapy regimen, is estimated to be approximately 20% [11–13]. The neutrophil support provided by efbemalenograstim alfa, a once per cycle G-CSF, was evaluated in this study and was demonstrated to have an efficacy and safety profile similar to pegfilgrastim.

Initially, this phase 2 open-label study admitted patients scheduled for treatment with TC chemotherapy; however, interim data demonstrated a lower rate of moderate and severe neutropenia than the rate of 43% observed by Bordoni et al. [14]. As a result, the study protocol was amended to admit patients undergoing TAC chemotherapy with the intent that this more rigorous regimen would provide more power to compare the efficacy of efbemalenograstim alfa with pegfilgrastim. Indeed, higher incidences of neutropenia with lower ANC nadir were observed with TAC chemotherapy, permitting adequate assessment of differences in the incidence and duration of neutropenia, and suggesting that TAC chemotherapy provided a better model for evaluating the efficacy of myeloid growth factors in this study.

In both myelotoxic chemotherapy regimens used in study, treatment with either 240 µg/kg or 320 µg/kg efbemalenograstim alfa was non-inferior to treatment with 6 mg pegfilgrastim in reducing the duration of moderate or severe neutropenia in the first cycle of chemotherapy. Efbemalenograstim alfa treatment in cycle 1 following TAC chemotherapy resulted in a duration of moderate and severe neutropenia of 2 days and 1.5 days. The duration of severe neutropenia observed in this study for pegfilgrastim (1.1 days) is slightly less than that seen in phase 3 studies with pegfilgrastim where the duration was 1.7–1.8 days; this variation is likely due to differences in study design and population [15–17]. Time to recovery post-nadir in this study was approximately 2 days, which is comparable to that seen in other studies [15–17].

The two highest doses of efbemalenograstim alfa that were examined in this dose-finding study, 240 and 320 µg/kg, showed similar effects on ANCs, while the lowest dose of efbemalenograstim alfa, 80 µg/kg, was less effective. For patients receiving TAC chemotherapy, the 80 µg/kg dose was not evaluated due to potential safety risks to patients. Pegfilgrastim is currently provided in a fixed dose of 6 mg, an approach that has several advantages over individualized dosing regimens and is associated with improved compliance, convenience, and decreased cost. The sponsor has completed three Phase III studies, in which the safety and efficacy of a fixed-dose administration of efbemalenograstim alfa, 20 mg PFS, equivalent to 320 µg/kg, have been demonstrated, making it a more convenient dosing option.

The overall safety profile of efbemalenograstim alfa was similar to that observed for pegfilgrastim 6 mg. AEs that occurred in this study have been observed in other studies involving G-CSFs, such as bone pain, or those associated with chemotherapy, such as alopecia and nausea. Occurrences of febrile neutropenia in this study were surprisingly low, with only 4 instances. The incidence of AEs, treatment-related AEs, AEs leading to discontinuation, injection site reactions, and febrile neutropenia were similar between the 320 µg/kg efbemalenograstim alfa and pegfilgrastim treatment arms. Compared to 320 µg/kg efbemalenograstim alfa, patients receiving pegfilgrastim had higher rates of SAEs and infections. With ANC < 1.0 × 10^9^/L, there is a substantial increased risk of infection [6, 18]. A dose-dependent trend was observed of decreasing incidence of infection (9%, 5%, and 3%) with increasing efbemalenograstim alfa dose (80, 240, and 320 µg/kg, respectively), further supporting efbemalenograstim alfa’s efficacy in preventing the negative clinical consequences of chemotherapy-induced hematological effects.

The interest in new G-CSF treatment options is evident in the number of such compounds that have recently been approved or are currently under investigation. In addition to various filgrastim biosimilars that have recently been approved [19–22], other novel G-CSFs are currently being investigated, including lipegfilgrastim, mecapegfilgrastim and eflapegrastim, which are designed to increase retention time and facilitate once-per-cycle dosing. Lipegfilgrastim, a glycoPEGylated G-CSF, was approved for use in the European Union in 2013 [18, 23, 24] while mecapegfilgrastim, a long-acting recombinant human G-CSF, was approved for use in China in 2018 [25]. Eflapegrastim, a long-acting G-CSF comprised of rhG-CSF covalently linked to human IgG4 Fc fragment via a PEG linker, was recently approved by FDA. It is the first novel long-acting G-CSF produced in over 20 years [26]. Similar to efbemalenograstim alfa, these compounds have demonstrated non-inferiority to pegfilgrastim based on the duration of severe neutropenia and have safety profiles similar to pegfilgrastim.

In addition to a larger molecular size to help reduce renal clearance, efbemalenograstim alfa was developed as a dimeric G-CSF to provide enhanced G-CSFR activation during chemotherapy. Conversely, the addition of PEG is known to reduce the affinity for receptor binding due to PEG’s hindering effect on binding to G-CSFR sites [7]. Preclinical studies have shown that efbemalenograstim alfa is a stronger activator of G-CSFR, based on faster neutrophil recovery times and the occurrence of less severe neutropenia; however, this has not yet been demonstrated in human clinical trials. It’s worth noting that eflapegrastim, rhG-CSF fused to human IgG4 Fc, demonstrated increased potency vs pegfilgrastim in preclinical studies and a Phase III trial [27, 28]. Unlike pegfilgrastim and filgrastim, which are primarily cleared via neutrophil-mediated and renal mechanisms, respectively [16], efbemalenograstim alfa and eflapegrastim clearance is thought to be mediated by G-CSFR which becomes saturated with high concentrations. Increasing the dosing schedule for efbemalenograstim alfa may lead to a clinically obvious manifestation of enhanced G-CSFR activation.

Unlike pegfilgrastim (Neulasta) and eflapegrastim (Rolvedon) which are derived from E. coli bacteria culture, efbemalenograstim alfa is produced in the Chinese hamster ovary cells (CHO) with modifications such as glycosylation that render its structure more like the original G-CSF, which may contribute to its increased potency in binding to receptors than those produced in bacteria.

In addition, in vitro studies showed that same-day administration of Eflagrastim with chemotherapy enhanced neutropenic recovery compared to pegfilgrastim in neutropenic rats [27]. Based on these observations, a Phase 1 clinical trial to evaluate safety and efficacy of same-day dosing of eflapegrastim in patients with breast cancer (NCT04187898) is ongoing. Results from the first 9 patients with early-stages breast cancer are very promising [29]. Evive’s in house studies in rats also showed assuring results of same-day administration of efbemalenograstim alfa (data not published).

Currently, all long-acting G-CSFs currently in the market are PEGylated G-CSFs including Rolvedon which was recently approved by FDA. Allergies to PEG are rare but increasingly recognized and can be severe. With the rising usage of PEG in commercial products, it has been reported that as many as 70% of people possess detectable levels of anti-PEG antibodies. Pre-existing anti-PEG antibodies may induce hypersensitivity reactions [7] while drug-induced PEG immunity may reduce the efficacy or safety of subsequently administered PEGylated drugs. Efbemalenograstim alfa is unique as a novel long-acting G-CSF without PEGylation.

Overall, the results from this study demonstrate that efbemalenograstim alfa is a safe and effective treatment for the management of neutropenia in patients undergoing myelosuppressive chemotherapy and is comparable to pegfilgrastim.

### Supplementary Information

Below is the link to the electronic supplementary material.

Supplementary file1 (DOC 277 KB)

## Data Availability

Not applicable.

## References

[1] Cooper KL, Madan J, Whyte S, Stevenson MD, Akehurst RL (2011). Granulocyte colony-stimulating factors for febrile neutropenia prophylaxis following chemotherapy: systematic review and meta-analysis. BMC Cancer.

[2] Lyman GH, Dale DC, Culakova E, Poniewierski MS, Wolff DA, Kuderer NM, Huang M, Crawford J (2013). The impact of the granulocyte colony-stimulating factor on chemotherapy dose intensity and cancer survival: a systematic review and meta-analysis of randomized controlled trials. Ann Oncol.

[3] Kojima S, Matsuyama T, Kodera Y, Nishihira H, Ueda K, Shimbo T, Nakahata T (1996). Measurement of endogenous plasma granulocyte colony-stimulating factor in patients with acquired aplastic anemia by a sensitive chemiluminescent immunoassay. Blood.

[4] Tamada T, Honjo E, Maeda Y, Okamoto T, Ishibashi M, Tokunaga M, Kuroki R (2006). Homodimeric cross-over structure of the human granulocyte colony-stimulating factor (GCSF) receptor signaling complex. Proc Natl Acad Sci USA.

[5] Bendall LJ, Bradstock KF (2014). G-CSF: from granulopoietic stimulant to bone marrow stem cell mobilizing agent. Cytokine Growth Factor Rev.

[6] Kozma GT, Shimizu T, Ishida T, Szebeni J (2020). Anti-PEG antibodies: properties, formation, testing and role in adverse immune reactions to PEGylated nano-biopharmaceuticals. Adv Drug Deliv Rev.

[7] Yang Q, Jacobs TM, McCallen JD, Moore DT, Huckaby JT, Edelstein JN, Lai SK (2016). Analysis of pre-existing IgG and IgM antibodies against polyethylene glycol (PEG) in the general population. Anal Chem.

[8] Hu Z, Huang ZH, Cen XB et al (2010) F-627, a G-CSF dimer, stimulated a more rapid neutrophil recovery in cyclophosphamide-treated monkeys compared to monomer rhG-CSFs. In 52nd ASH annual meeting and exposition

[9] Rapoport BL, Garcia-Morillo M, Font C, Samoon Z (2023). A prospective, real-world, multinational study of febrile neutropenia (FN) occurrence in oncology patients receiving chemotherapy with intermediate risk of FN: a MASCC Neutropenia, Infection, and Myelosuppression Study Group initiative. Support Care Cancer.

[10] Fontanella C, Bolzonello S, Lederer B, Aprile G (2014). Management of breast cancer patients with chemotherapy-induced neutropenia or febrile neutropenia. Breast Care (Basel).

[11] Smith TJ, Khatcheressian J, Lyman GH (2006). 2006 update of recommendations for the use of white blood cell growth factors: an evidence-based clinical practice guideline. J Clin Oncol.

[12] Fagnani D, Isa L, Verga MF (2014). Granulocyte colony-stimulating factors used in clinical practice: PoloNord Registry-Based Cohort Italian Study. Tumori.

[13] Rigacci L, Puccini B, Kovalchuk S, Fabbri E, Bonizzoni E, Perrone T, Bosi A (2014). Feasibility and safety of a reduced duration of therapy of colony-stimulating factor in a dose-dense regimen. Support Care Cancer.

[14] Bordoni RE, Haislip ST, Gilmore JW, Sharpe J, Choi MR, Abella E (2012). Estimation of the incidence of febrile neutropenia in women receiving docetaxel plus cyclophosphamide as adjuvant therapy for early-stage breast cancer: a large community-based retrospective study. Commun Oncol.

[15] Holmes FA, Jones SE, O’Shaughnessy J, Vukelja S, George T, Savin M, Richards D, Glaspy J, Meza L, Cohen G, Dhami M, Budman DR, Hackett J, Brassard M, Yang BB, Liang BC (2002). Comparable efficacy, and safety profiles of once-per-cycle pegfilgrastim and daily injection filgrastim in chemotherapy-induced neutropenia: a multicenter dose-finding study in women with breast cancer. Ann Oncol.

[16] Green MD, Koelbl H, Baselga J, Galid A, Guillem V, Gascon P, Siena S, Lalisang RI, Samonigg H, Clemens MR, Zani V, Liang BC, Renwick J, Piccart MJ, International Pegfilgrastim 749 Study Group (2003). A randomized double-blind multicenter phase III study of fixed-dose single-administration pegfilgrastim versus daily filgrastim in patients receiving myelosuppressive chemotherapy. Ann Oncol.

[17] Lyman GH (2005). Pegfilgrastim: a granulocyte colony-stimulating factor with sustained duration of action. Expert Opin Biol Ther.

[18] Bondarenko I, Gladkov OA, Elsaesser R, Buchner A, Bias P (2013). Efficacy and safety of lipegfilgrastim versus pegfilgrastim: a randomized, multicenter, active-control phase 3 trial in patients with breast cancer receiving doxorubicin/docetaxel chemotherapy. BMC Cancer.

[19] FULPHILA [Prescribing Information] (2018) Zurich, Switzerland: Mylan GmbH

[20] NIVESTYM [Prescribing Information] (2018) Illinois, United States: Hospira, Inc

[21] ZIEXTENZO [Prescribing Information] (2019) New Jersey, United States: Sandoz, Inc

[22] NYVEPRIA [Prescribing Information] (2020) Illinois, United States: Hospira, Inc

[23] Buchner A, Elsässer R, Bias P (2014). A randomized, double-blind, active control, multicenter, dose-finding study of lipegfilgrastim (XM22) in breast cancer patients receiving myelosuppressive therapy. Breast Cancer Res Treat.

[24] Lonquex [Summary of Product Characteristics] (2013) Haarlem, Netherlands: Teva Biotech GmbH

[25] Xu F, Zhang Y, Miao Z, Zeng X, Wu B, Cai L, Liu J, Wang S, Hu X, Zheng W, Chen Z, Yang Q, Jiang Z (2019). Efficacy and safety of mecapegfilgrastim for prophylaxis of chemotherapy-induced neutropenia in patients with breast cancer: a randomized, multicenter, active-controlled phase III trial. Ann Transl Med.

[26] ROLVEDON [Prescribing Information] (2022) California, United States: Spectrum Pharmaceuticals, Inc

[27] Barrett JA, Choi J, Lakshmikanthan S, Kim YY, Greene D, Kolli P, Song TH, Choi IY, Kim YH, Lebel F (2020). Eflapegrastim’s enhancement of efficacy compared with pegfilgrastim in neutropenic rats supports potential for same-day dosing. Exp Hematol.

[28] Schwartzberg LS, Bhat G, Peguero J, Agajanian R, Bharadwaj JS, Restrepo A, Hlalah O, Mehmi I, Chawla S, Hasal SJ, Yang Z, Cobb PW (2020). Eflapegrastim, a long-acting granulocyte-colony stimulating factor for the management of chemotherapy-induced neutropenia: results of a phase III trial. Oncologist.

[29] Schwartzberg LS, Francis J, Osama H, Modiano M, Bharadwaj J, Chawla S, Bhat G, Lebel F, Tchekmedyian N (2020). Open-label, phase 1 study to evaluate duration of severe neutropenia after same-day dosing of eflapegrastim in patients with breast cancer receiving docetaxel and cyclophosphamide (NCT04187898). Blood.

