# Supplementary material for: Efbemalenograstim alfa not inferior to pegfilgrastim in providing neutrophil support in women with breast cancer undergoing myelotoxic chemotherapy: results of a phase 2 randomized, multicenter, open-label trial
Source: Support Care Cancer. 2024 Jan 9;32(2):91. doi: 10.1007/s00520-023-08260-x (PMC10776461; doi:10.1007/s00520-023-08260-x)
Supplement: Supplementary file 1 — Supplementary file1 (DOC 277 KB) [file 520_2023_8260_MOESM1_ESM.doc]

List of Online Resources

[Online Resource 1 Patient Flow for TC Chemotherapy Population 4](#__RefHeading___Toc56504502)

[Online Resource 2 Baseline Demographic and Disease Status for Patients in TC Chemotherapy Population 5](#__RefHeading___Toc56504503)

[Online Resource 3 Duration of Neutropenia in Chemotherapy Cycle 1 for TC Chemotherapy Population 6](#__RefHeading___Toc56504504)

[Online Resource 4 Mean Absolute Neutrophil Count During All TAC Chemotherapy Cycles 7](#__RefHeading___Toc56504505)

[Online Resource 5 Duration of Moderate and Severe Neutropenia in Chemotherapy Cycles 2-4 8](#__RefHeading___Toc56504506)

[Online Resource 6 Incidence of Neutropenia in TAC Chemotherapy Cycles 2 to 4 9](#__RefHeading___Toc56504507)

**
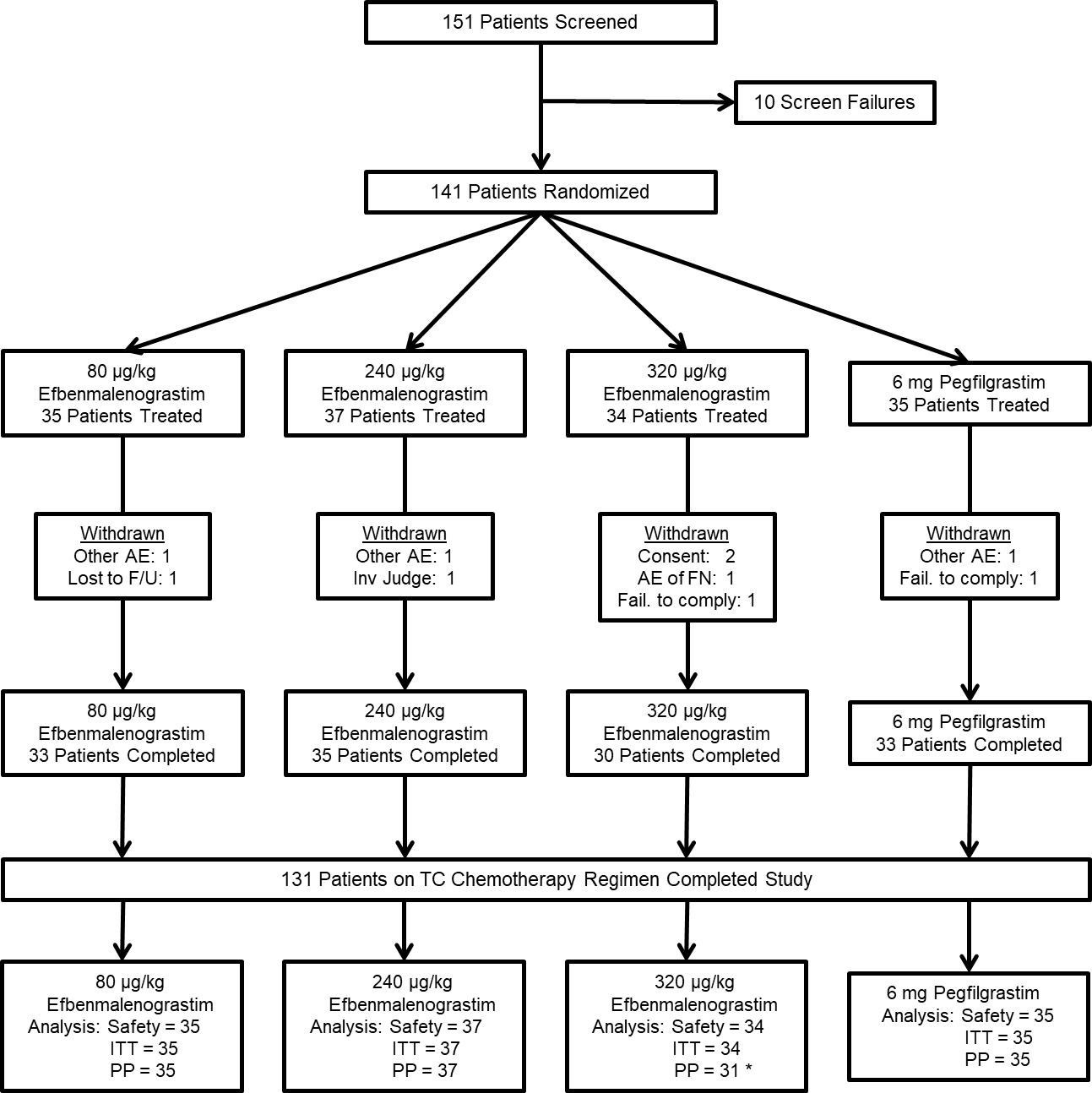
**

Online Resource 1 Patient Flow for TC Chemotherapy Population

AE = adverse event; FN = febrile neutropenia; ITT = Intent-to-Treat; PP = Per Protocol; TC = Taxotere® [docetaxel] + cyclophosphamide.

* 2 patients were excluded from the PP population due to lack of absolute neutrophil count data in chemotherapy cycle 1 and 1 patient was excluded due to early discontinuation.

| Online Resource 2 Baseline Demographic and Disease Status for Patients in TC Chemotherapy Population | | | | |
| --- | --- | --- | --- | --- |
|  | Efbenmalenograstim | | | Pegfilgrastim 6 mg/dose N = 35 |
|  | 80 μg/kg/dose  N = 35 | 240 μg/kg/dose  N = 37 | 320 μg/kg/dose  N = 34 |  |
| **Age, mean (range), years** | 48.7 (25, 73) | 47.6 (30, 74) | 51.2 (28, 68) | 48.2 (18, 65) |
| **Reproductive Status** |  |  |  |  |
| Childbearing potential | 20 (57.1) | 21 (56.8) | 14 (41.2) | 18 (51.4) |
| Non-childbearing potential | 15 (42.9) | 16 (43.2) | 20 (58.8) | 17 (48.6) |
| **Race** |  |  |  |  |
| Caucasian | 35 (100.0) | 36 (97.3) | 33 (97.1) | 35 (100.0) |
| Black/African American | 0 | 0 | 1 (2.9) | 0 |
| Others | 0 | 1 (2.7) | 0 | 0 |
| **Country/Region** |  |  |  |  |
| Ukraine | 13 (37.1) | 14 (37.8) | 13 (38.2) | 14 (40.0) |
| Russia | 19 (54.3) | 19 (51.4) | 19 (55.9) | 19 (54.3) |
| USA | 3 (8.6) | 4 (10.8) | 2 (5.9) | 2 (5.7) |
| **ECOG Performance Status** |  |  |  |  |
| 0 | 25 (71.4) | 20 (54.1) | 20 (58.8) | 24 (68.6) |
| 1 | 10 (28.6) | 17 (45.9) | 14 (41.2) | 11 (31.4) |
| **Cancer Stage at Screening** |  |  |  |  |
| I | 1 (2.9) | 8 (21.6) | 7 (20.6) | 4 (11.4) |
| II | 25 (71.4) | 15 (40.5) | 15 (44.1) | 21 (60.0) |
| III | 9 (25.7) | 14 (37.8) | 12 (35.3) | 10 (28.6) |
| IV | 0 | 0 | 0 | 0 |
| Prior Surgery for Breast Cancer | 30 (85.7) | 34 (91.9) | 31 (91.2) | 27 (77.1) |
| Prior Systemic Therapy | 2 (5.7) | 4 (10.8) | 2 (5.9) | 1 (2.9) |
| Prior Radiation Therapy | 4 (11.4) | 5 (13.5) | 5 (14.7) | 2 (5.7) |
| Data presented as n (%) unless otherwise indicated.  ECOG = Eastern Cooperative Oncology Group; TC = Taxotere® [docetaxel] + cyclophosphamide. | | | | |

| Online Resource 3 Duration of Neutropenia in Chemotherapy Cycle 1 for TC Chemotherapy Population | | | | |
| --- | --- | --- | --- | --- |
|  | Efbenmalenograstim | | | Pegfilgrastim 6 mg N = 35 |
|  | 80 µg/kg N = 35 | 240 µg/kg N = 37 | 320 µg/kg N = 31 |
| **Moderate and Severe Neutropenia (ANC <1.0×10^9^/L)** | | | | |
| Duration, days |  |  |  |  |
| Mean (SD) | 0.6 (1.26) | 0.6 (1.01) | 0.4 (0.75) | 0.3 (0.56) |
| Median (range) | 0.0 (0, 5) | 0.0 (0, 3) | 0.0 (0, 2) | 0.0 (0, 2) |
| Difference vs. pegfilgrastim (95% CI) | 0.4 (–0.1, 0.8) | 0.3 (–0.1, 0.7) | 0.1 (–0.4, 0.6) |  |
| Non-inferior to pegfilgrastim? | Yes | Yes | Yes |  |
| Superior to pegfilgrastim? | No | No | No |  |
| **Severe Neutropenia (ANC <0.5×10^9^/L)** | | | | |
| Duration, days |  |  |  |  |
| Mean (SD) | 0.2 (0.73) | 0.4 (0.86) | 0.2 (0.48) | 0.1 (0.28) |
| Median (range) | 0.0 (0, 3) | 0.0 (0, 3) | 0.0 (0, 2) | 0.0 (0, 1) |
| Difference vs. pegfilgrastim (95% CI) | 0.1 (–0.2, 0.4) | 0.3 (0.0, 0.6) | 0.1 (–0.2, 0.4) |  |
| ANC = absolute neutrophil count; CI = confidence interval; SD = standard deviation; TC = Taxotere® [docetaxel] + cyclophosphamide. | | | | |


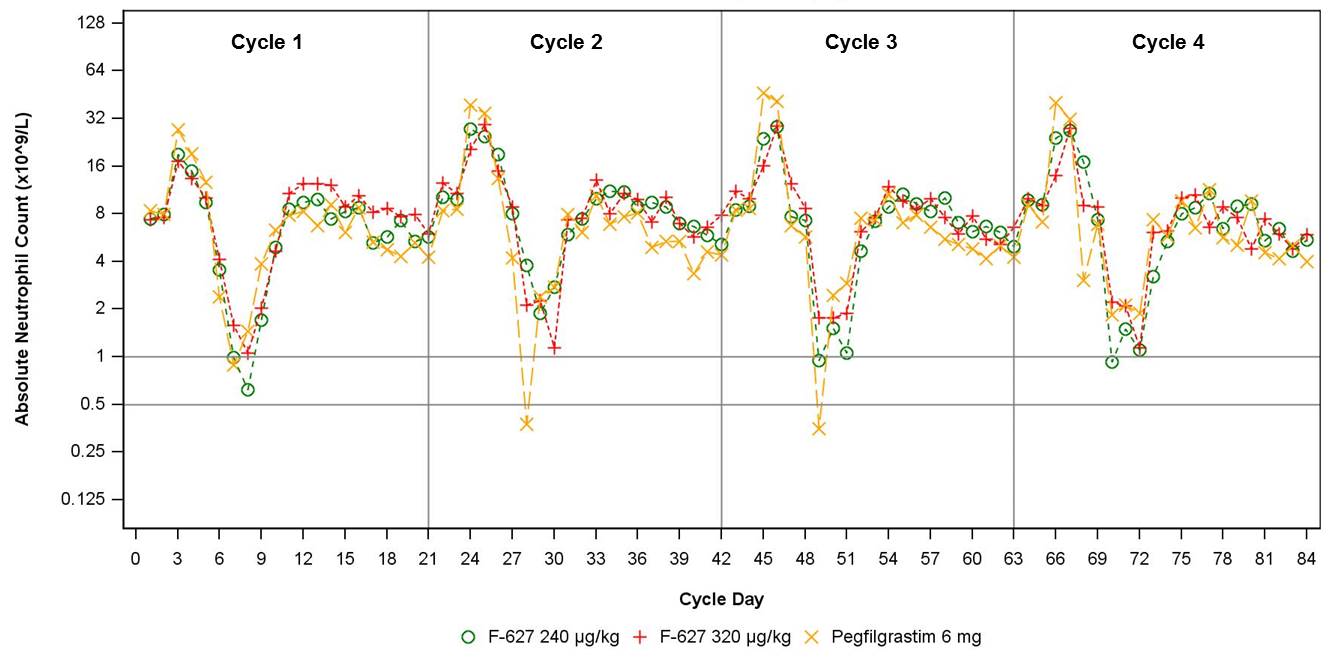


Online Resource 4 Mean Absolute Neutrophil Count During All TAC Chemotherapy Cycles

F-627 = Efbenmalenograstim; TAC = Taxotere® [docetaxel] + doxorubicin + cyclophosphamide.

| Online Resource 5 Duration of Moderate and Severe Neutropenia in Chemotherapy Cycles 2-4 | | | | | | | | |  |
| --- | --- | --- | --- | --- | --- | --- | --- | --- | --- |
|  | TAC Chemotherapy Population | | |  | TC Chemotherapy Population | | | | |
|  | Efbenmalenograstim | | Pegfilgrastim 6 mg |  | Efbenmalenograstim | | | Pegfilgrastim 6 mg | |
|  | 240 µg/kg | 320 µg/kg |  |  | 80 µg/kg | 240 µg/kg | 320 µg/kg |  |  |
| Cycle 2 | N = 27 | N = 28 | N = 28 |  | N = 34 | N = 35 | N = 30 | N = 34 | |
| Mean (SD) duration, days | 0.9 (1.21) | 1.2 (1.61) | 0.9 (1.31) |  | 0.3 (1.17) | 0.1 (0.47) | 0.1 (0.51) | 0.0 (0.00) | |
| Difference vs. pegfilgrastim (95% CI) | 0.0, (–0.7, 0.8) | 0.3 (–0.5, 1.0) |  |  | 0.3 (0.0, 0.7) | 0.1 (–0.2, 0.4) | 0.1 (–0.2, 0.5) |  | |
| Cycle 3 | N = 27 | N = 28 | N = 27 |  | N = 34 | N = 34 | N = 29 | N = 34 | |
| Mean (SD) duration, days | 1.2 (1.67) | 1.3 (1.40) | 0.6 (1.08) |  | 0.3 (0.94) | 0.1 (0.48) | 0.1 (0.37) | 0.1 (0.34) | |
| Difference vs. pegfilgrastim (95% CI) | 0.6 (–0.1, 1.4) | 0.7 (–0.1, 1.4) |  |  | 0.3 (0.0, 0.5) | 0.1 (–0.2, 0.3) | 0.0 (–0.3, 0.3) |  | |
| Cycle 4 | N = 25 | N = 28 | N = 27 |  | N = 34 | N = 34 | N = 29 | N = 33 | |
| Mean (SD) duration, days | 1.6 (1.73) | 1.5 (1.64) | 0.8 (1.72) |  | 0.4 (1.18) | 0.2 (0.84) | 0.0 (0.00) | 0.0 (0.00) | |
| Difference vs. pegfilgrastim (95% CI) | 0.8 (–0.2, 1.7) | 0.8 (–0.2, 1.7) |  |  | 0.4 (0.1, 0.8) | 0.2 (–0.2, 0.6) | 0.0 (–0.4, 0.4) |  | |
| CI = confidence interval; SD = standard deviation; TAC = Taxotere® [docetaxel] + doxorubicin + cyclophosphamide; TC = Taxotere® [docetaxel] + cyclophosphamide. | | | | | | | | |  |

| Online Resource 6 Incidence of Neutropenia in TAC Chemotherapy Cycles 2 to 4 | | | |
| --- | --- | --- | --- |
|  | Efbenmalenograstim | | Pegfilgrastim 6 mg |
|  | 240 µg/kg | 320 µg/kg |
| **Cycle 2** | | | |
| ANC <1.0 x 10^9^/L, n/N (%) | 11/27 (40.7) | 12/28 (42.9) | 10/28 (35.7) |
| Difference vs. pegfilgrastim (*P*)^a^ | 5.0 (0.7848) | 7.1 (0.7848) |  |
| ANC <0.5 x 10^9^/L, n/N (%) | 7/27 (25.9) | 3/28 (10.7) | 5/28 (17.9) |
| Difference vs. pegfilgrastim (*P*)^a^ | 8.1 (0.5279) | -7.1 (0.7049) |  |
| Cycle 3 |  |  |  |
| ANC <1.0 x 10^9^/L, n/N (%) | 12/27 (44.4) | 14/28 (50.0) | 7/27 (25.9) |
| Difference vs. pegfilgrastim (*P*)^a^ | 18.5 (0.2542) | 24.1 (0.0966) |  |
| ANC <0.5 x 10^9^/L, n/N (%) | 5/27 (18.5) | 6/28 (21.4) | 4/27 (14.8) |
| Difference vs. pegfilgrastim (*P*)^a^ | 3.7 (1.0000) | 6.6 (0.7287) |  |
| Cycle 4 |  |  |  |
| ANC <1.0 x 10^9^/L, n/N (%) | 14/25 (56.0) | 15/28 (53.6) | 6/27 (22.2) |
| Difference vs. pegfilgrastim (*P*)^a^ | 33.8 (0.0217) | 31.3 (0.0261) |  |
| ANC <0.5 x 10^9^/L, n/N (%) | 5/25 (20.0) | 8/28 (28.6) | 6/27 (22.2) |
| Difference vs. pegfilgrastim (*P*)^a^ | -2.2 (1.0000) | 6.3 (0.7585) |  |
| ANC = absolute neutrophil count; TAC = Taxotere® [docetaxel] + doxorubicin + cyclophosphamide.  ^a^ Fisher’s Exact test. | | | |
